#  Are the effects of nicotinic acid on insulin resistance precipitated by abnormal phosphorous metabolism?

**DOI:** 10.1186/1476-511X-3-23

**Published:** 2004-10-28

**Authors:** Moutasim H Al-Shaer, Hatem S AbuSabha

**Affiliations:** 1 The Department of Internal Medicine and Human Cardiovascular Physiology Laboratory University of Iowa College of Medicine Iowa City, Iowa 52242-1009, USA

**Keywords:** Phosphorous, Nicotinic acid, Insulin resistance, carbohydrate metabolism, diabetes, metabolic syndrome, Dyslipidemia, Hyperlipidemia, Hypercholesterolemia

## Abstract

Nicotinic acid is a unique cholesterol modifying agent that exerts favorable effects on all cholesterol parameters. It holds promise as one of the main pharmacological agents to treat mixed dyslipidemia in metabolic syndrome and diabetic patients. The use of nicotinic acid has always been haunted with concerns that it might worsen insulin resistance and complicate diabetes management.

We will discuss the interaction between phosphorous metabolism and carbohydrate metabolism and the possibility that worsening of insulin resistance could be related to adrug induced alteration in phosphorous metabolism, and the implications of that in medical management of diabetes and metabolic syndrome patients with mixed dyslipidemia.

## Background

Nicotinic acid functions in the body after conversion to nicotinamide adenine dinucleotide (NAD) in the NAD coenzyme system. Niacin reduces total cholesterol (TC), low-density lipoprotein cholesterol (LDL-C), triglycerides (TG), apolipoprotein B-100 (Apo B) and Lipoprotein (a) (Lp (a)). Niacin increases high-density lipoprotein cholesterol (HDL-C), apolipoprotein A-I (Apo A-I), lipoprotein A-I, and HDL2: HDL3 ratio. The severity and type of underlying lipid abnormality determines the extent of response to niacin treatment [1].

There is evidence suggesting that niacin reduces the risk of a coronary artery or cerebrovascular disease in the setting of secondary prevention, and when combined with a bile acid-sequestrant, promotes regression and decreases progression of atherosclerotic cardiovascular disease [2,3].

Niacin is known to cause transient, small but statistically significant dose-related reductions in phosphorous levels. These effects were studied for extended release niacin in placebo-controlled trials, mean percentage changes from baseline (± standard error) in phosphorous were (-4.0 ± 1.3) for the 500 mg once daily dose, (-7.2 ± 1.1) for the 1000 mg once daily dose, (-9.1 ± 1.1) for the 1500 mg once daily dose, and (-12.6 ± 1.6) for the 2000 mg once daily dose [4].

## Presentation of the hypothesis

It has been long known that niacin induces impaired fasting glucose. I was also suggested that this effect is most likely transient. The mechanism behind this phenomenon has not been fully elucidated [5].

Multiple studies were conducted to study effects of nicotinic acid on exacerbating insulin resistance. One retrospective study suggested that the use of moderate doses of extended release nicotinic acid (average, 1000 mg/d) in reasonably controlled diabetics was associated with improved glycemic control with HbA1c levels decreased by 0.5% ± 0.3% due to aggressive hypoglycemic therapy; as most of these patients had their insulin or oral diabetes regimen intensified [6]. Another retrospective study using unmodified niacin concluded similar results [7].

The (ADMIT) trial, was one of the first studies to demonstrate the safety of nicotinic acid in patients with diabetes. Diabetic patients (468 participants, 125 with diabetes and peripheral arterial disease) were randomized to treatment with either placebo or niacin (2500 mg/d) for 18 weeks. Patients randomized to niacin therapy had modest increases in fasting glucose (+8 mg/dL). There was no significant change in HbA1c levels or in diabetes treatment regimen in the niacin-treated diabetic patients group [8]. Another prospective Trial (ADVENT), was a 16-week, double-blind, placebo-controlled trial, 148 patients were randomized to placebo (n = 49) or 1000 (n = 45) or 1500 mg/d (n = 52) of extended release niacin. Fasting plasma glucose levels in both treatment groups were unchanged from placebo at the study's end. Meanwhile, the mean HbA1c levels were only slightly elevated in the 1500 mg arm, compared with placebo. The 1500 mg nicotinic acid arm required a small increase in anti-diabetic treatment regimen [9].

It appears from the clinical data presented above, that nicotinic acid at higher doses could results in alterations in glycemic control of patients with an already abnormal glucose metabolism.

Phosphate is primarily an intracellular anion. Insulin is implicated in the transport of glucose and phosphate from the intra-cellular to the extra-cellular space. Studies have suggested a link between decreased serum phosphorous and disturbed carbohydrate metabolism, eventually leading to hyperglycemia [10,11]. Chronic hypophosphatemia inhibits glucose transport. Further more, reduced serum phosphorous levels significantly decrease the phosphorylation of carbohydrate intermediates in glycolysis and glycogenesis.

It seems intuitive to suspect that the transient decrease in plasma phosphorous level might explain the transient disturbance in carbohydrate metabolism and hyperglycemia induced by nicotinic acid. Also, studies suggest the existence of a dose response relationship in both instances.

One way to test this hypothesis will be to administer nicotinic acid to both healthy subjects, and to those with impaired fasting glucose or subjects with diabetes, then measure simultaneously at different doses serum phosphorous, and correlate the changes to changes in serum glucose and serum insulin either individually or combined as a measure of insulin resistance.

It will be of interest to study if such a relationship exists in patients with the metabolic syndrome diagnosis, since literature suggests the existence of an abnormal phosphorous metabolism in these patients [10,12].

## Implications of the hypothesis

This hypothesis has several important insights and implications:

1. If valid, it suggests the possibility of a simple clinical way to prevent nicotinic acid induced worsening of glycemic control by dietary phosphorous supplementation, eliminating the need to intensify expensive diabetic regimens in diabetic patients that need nicotinic acid treatment.

2. If valid, it will aid in lessening the theoretical anxiety of the possibility of precipitating diabetes in glucose intolerant or metabolic syndrome patients in need of nicotinic acid treatment for the management of dyslipidemia.

3. Metabolic syndrome and diabetics stand to benefit greatly from the new evidence implicating the importance of increasing HDL-cholesterol in cardiovascular event reduction.

4. This hypothesis offers new insights into the mechanism of development of glucose intolerance.

## References

[B1] (2004). Physician desk reference (PDR).

[B2] Cashin-Hemphill L, Mack WJ, Pogoda JM, Sanmarco ME, Azen SP, Blankenhorn DH (1990). Beneficial effects of colestipol-niacin on coronary atherosclerosis. A 4-year follow-up. JAMA.

[B3] The Coronary Drug Project Research Group (1975). Clofibrate and niacin in coronary heart disease. JAMA.

[B4] Data on file. Kos Pharmaceuticals, Inc.

[B5] Meyers CD, Kashyap ML (2004). Management of the metabolic syndrome-nicotinic acid. Endocrinol Metab Clin North Am.

[B6] Kane MP, Hamilton RA, Addesse E, Busch RS, Bakst G (2001). Cholesterol and glycemic effects of Niaspan in patients with type 2 diabetes. Pharmacotherapy.

[B7] Pan J, Lin M, Kesala RL, Van J, Charles MA (2002). Niacin treatment of the atherogenic lipid profile and Lp(a) in diabetes. Diabetes Obes Metab.

[B8] Elam MB, Hunninghake DB, Davis KB, Garg R, Johnson C, Egan D, Kostis JB, Sheps DS, Brinton EA (2000). Effect of niacin on lipid and lipoprotein levels and glycemic control in patients with diabetes and peripheral arterial disease: the ADMIT study: A randomized trial. Arterial Disease Multiple Intervention Trial.. JAMA.

[B9] Grundy SM, Vega GL, McGovern ME, Tulloch BR, Kendall DM, Fitz-Patrick D, Ganda OP, Rosenson RS, Buse JB, Robertson DD, Sheehan JP, Diabetes Multicenter Research Group (2002). Efficacy, safety, and tolerability of once-daily niacin for the treatment of dyslipidemia associated with type 2 diabetes: results of the assessment of diabetes control and evaluation of the efficacy of niaspan trial. Arch Intern Med.

[B10] Haglin L (2001). Hypophosphataemia: cause of the disturbed metabolism in the metabolic syndrome. Med Hypotheses.

[B11] Guyton AC, Hall JE Metabolism and temperature regulation in Textbook of Medical Physiology.

[B12] Haglin L, Lindblad A, Bygren LO (2001). Hypophosphataemia in the metabolic syndrome. Gender differences in body weight and blood glucose. Eur J Clin Nutr.

